# Using a 3D asymmetry index as a novel form for capturing complex three-dimensionality in positional plagiocephaly

**DOI:** 10.1038/s41598-022-24555-1

**Published:** 2022-12-02

**Authors:** Christian Linz, Julian Faber, Reiner Schmid, Felix Kunz, Hartmut Böhm, Stefan Hartmann, Tilmann Schweitzer

**Affiliations:** 1 Department of Oral and Maxillofacial Plastic Surgery, University Hospital Würzburg, Pleicherwall 2, 97070 Würzburg, Germany; 2 Department of Orthodontics, University Hospital Würzburg, Pleicherwall 2, 97070 Würzburg, Germany; 3 Section of Pediatric Neurosurgery, Department of Neurosurgery, University Hospital Würzburg, Josef-Schneider-Straße 2, 97070 Würzburg, Germany

**Keywords:** Craniofacial orthodontics, Physical examination, Three-dimensional imaging

## Abstract

Positional plagiocephaly (PP) is the most common skull deformity in infants. Different classification systems exist for graduating the degree of PP, but all of these systems are based on two-dimensional (2D) parameters. This limitation leads to several problems stemming from the fact that 2D parameters are used to classify the three-dimensional (3D) shape of the head. We therefore evaluate existing measurement parameters and validate a newly developed 3D parameter for quantifying PP. Additionally, we present a new classification of PP based on a 3D parameter. 210 patients with PP and 50 patients without PP were included in this study. Existing parameters (2D and 3D) and newly developed volume parameters based on a 3D stereophotogrammetry scan were validated using ROC curves. Additionally, thresholds for the new 3D parameter of a *3D asymmetry index* were assessed. The volume parameter *3D asymmetry index* quantifies PP equally as well as the gold standard of *30° diagonal difference*. Moreover, a *3D asymmetry index* allows for a 3D-based classification of PP. The *3D asymmetry index* can be used to define the degree of PP. It is easily applicable in stereophotogrammetric datasets and allows for comparability both intra- and inter-individually as well as for scientific analysis.

## Introduction

Positional plagiocephaly (PP) is the most common form of skull deformity in infants. PP maintains a typical parallelogram appearance in the vertex view of the infant’s skull that includes unilateral occipital flattening with ipsilateral anterior ear shift and ipsilateral frontal bossing.

Reports on the incidence of PP vary from 0.3 to 47%^1–3^. This immense variability in the percentage of incidence is due—inter alia—to the use of different classification systems or measurement methods^4^.

Argenta et al. proposed a classification based on clinical appearance^5^. To quantify Argenta’s description, different two-dimensional (2D) linear- and three-dimensional (3D) volume-based measurement methods have been employed.

2D measurements with direct cephalometry are performed by calliper and measuring tape using defined anatomical landmarks as references^6–9^. Restricted compliance by the infants causes some susceptibility to errors^10–12^. Moreover, capturing the inherently 3D nature of the shape of the head using less complex 2D capturing has several limitations, which include movement artefacts and insufficient transformation^13,14^.

Moss’s classification—which was supplemented by Mortenson et al.—is based on 2D measurements^6,11^. Both classification systems are based on so-called *cranial vault asymmetry* (*CVA*), which is generated by calculating the difference between the distances from the fronto-zygoma (the most lateral point of the fronto-zygomatic suture) and the diagonal eurion (the lateral point of the greatest width of the base of the skull) on each side^1,6,15^. Loveday et al. modified this CVA by defining the occipital measurement point of these diagonal lines in a fixed angle with 30° deviation from the median sagittal plane because a defined angle enhances reproducibility^10^. Additionally, Loveday et al. ended up with a percentage value because they had defined a relation between skull asymmetry and skull size^10^. Although the above-depicted classifications have widespread clinical application, they have yet to be further validated^16^.

3D stereophotogrammetry—the gold standard—is a form of non-invasive three-dimensional imaging. It is a radiation-free modality that precisely depicts surface structures without movement artifacts^17,18^ and is specified, validated, and regarded as reliable^18–21^. Although 3D stereophotogrammetry captures volumetric data, no classification yet exists that encompasses the complex 3D nature of head shape.

Therefore, the scope of our report lies in critically subsuming the established measurement parameters (2D and 3D) as well as in validating a newly developed 3D parameter for quantifying PP.

## Methods and material

In the present study, we investigated children with and without PP. The current study was based on a retrospective design using a standard measurement protocol, examined and approved by the Internal Review Board (Ethics Committee) of the University of Würzburg (143/09). The study was carried out according to the Declaration of Helsinki and written informed consent was obtained from the parents. Inclusion criteria for the children of both the control group and patient group were a complete 3D stereophotogrammetry scan and subsequent analysis (see data analysis) as well as manually performed cephalometry on the same day. Exclusion criteria were children with a brachycephalic head shape indicated by a cranial index (CI) ≥ 94%, any congenital anomaly or complications at delivery or postpartum including a stay at ICU^22^.

In line with the criteria defined by Moss and Mortenson et al., the infants` head shapes were categorised using a measurement along a defined 30° diagonal, as described by Loveday et al.^6,10^. A CVA of < 3 mm was classified as a *physiological* form of the skull, an asymmetry of ≥ 3 mm and ≤ 12 mm was classified as a moderate form of PP (*mild to moderate*), and an asymmetry of > 12 mm was classified as a severe (*moderate to severe*) form of PP (see Table 1)^6,10,11^.

**Table 1:** Classification of PP using CVA (at 30°).

CVA	Form
< 3 mm	Physiological
> 3 mm and < 12 mm	Moderate PP
> 12 mm	Severe PP

### Participants

#### Patients

210 patients with unilateral PP were included in the study, which covered a mean of 38.1 ± 2.7 weeks of gestation (WOG). Mean patient age was 6.5 ± 1.9 months. As reliable and manually performed cephalometry was not achievable in five infants, the number of patients in the parameter of *asymmetry* was reduced to n = 205.

**Figure 1:**
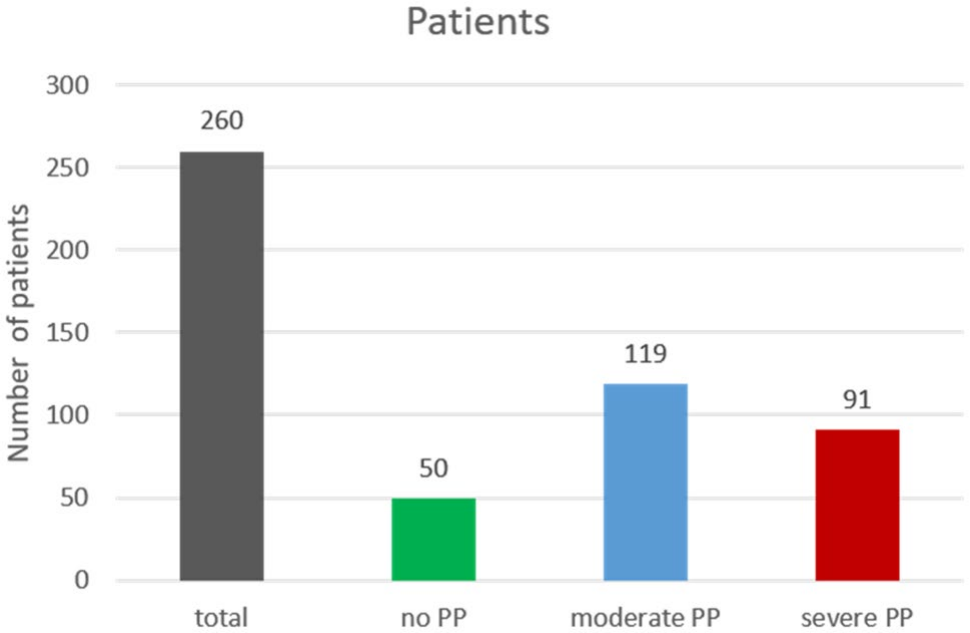
Number of patients.

According to the criteria defined by Moss and Mortenson et al., 119 infants showed moderate PP with a CVA (at 30°) of 0.9 ± 0.2 cm, while 91 infants demonstrated severe PP with a CVA of 1.6 ± 0.3 cm (Fig. 1).

#### Control

The control group consisted of n = 50 infants with a mean age of 6.4 ± 0.6 months. Mean WOG were 39.7 ± 1.3. Mean CVA was 0.2 ± 0.1 cm (Fig. 1.).

### Treatment protocol

As part of the first referral, an experienced physician examined all infants. Manually generated cephalometric measurements using a precision calliper and a measuring tape were carried out with a well-established examination protocol. 3D stereophotogrammetric data were acquired during the clinical routine workup using a 3dMD®-scanner with five synchronised cameras (3dMD, Atlanta, USA). Acquisition time is 1.5 ms with a linear accuracy range of 0.2 mm or better and high-quality texture maps that exceed full-HD densities.

### 3D stereophotogrammetry (data acquisition)

To generate a coordinate system for use in evaluating the resulting data, we applied established reference points, which are easily depictable in anatomical structures on the skull surface and were integrated using the VAM software (‘Visualization, Analysis, Measurement’, Fairfield, USA, Vectra). Parameters in this generated coordinate system can be measured using special analysis software (Cranioform Analytics^®^ 4.0; Cranioform, Alpnach, Switzerland).

**Figure 2:**
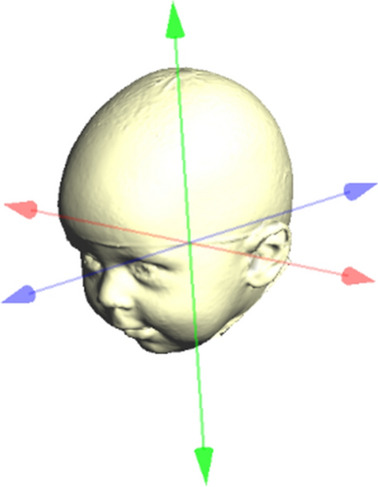
Coordinate system. x-axis: red; y-axis: blue; z-axis: green.

The middle of the line segment between the two tragus points (TrR/TrL) defines the centre (midpoint, M) of the coordinate system. The y-axis is set up on the connection between the centre (M) and the nasion point (N). In the first step in defining the x-axis, an auxiliary plane—defined by points M, N, and Sn (subnasale)—is needed. The x-axis then corresponds to the normal vector starting from point M. Finally, the z-axis is created by means of a straight line starting from point M that is perpendicular to the other two axes of the coordinate system. The coordinate system is shown in Fig. 2.

**Figure 3:**
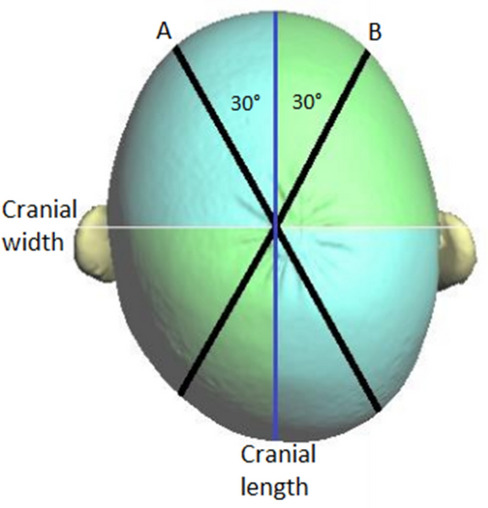
30° diagonals.

**Figure 4:**
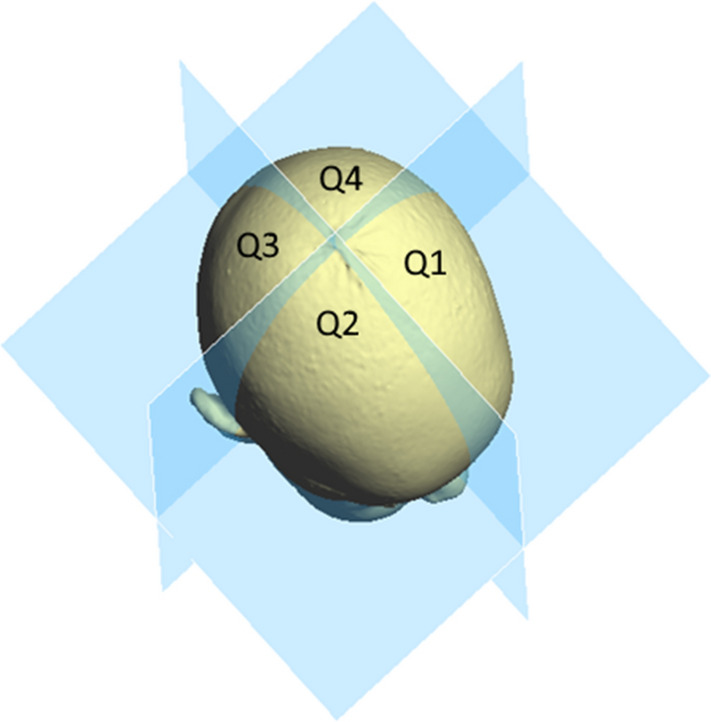
The four volume quadrants.

**Figure 5:**
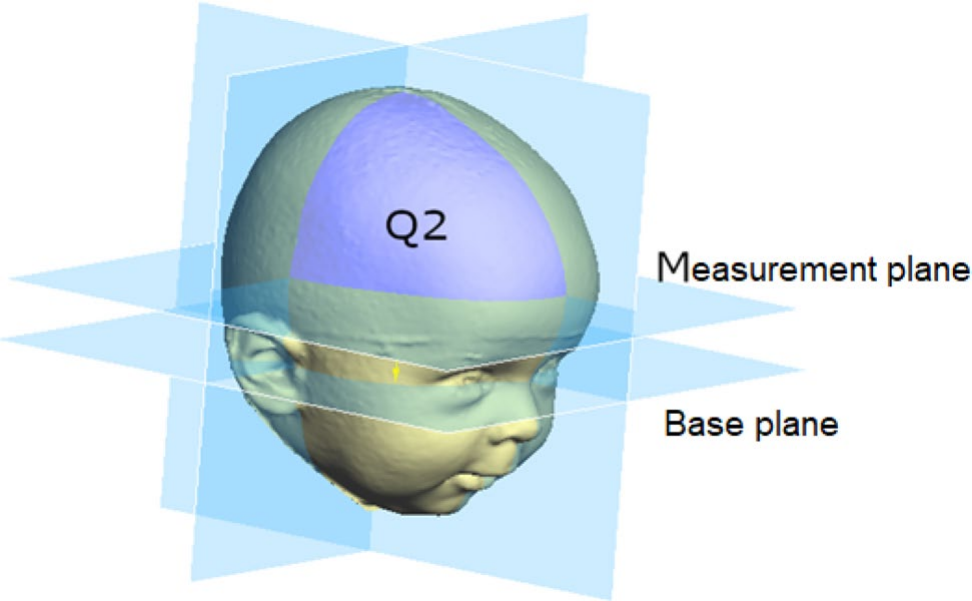
Measurement plane.

The corresponding planes are the base plane (horizontal), the auxiliary plane (sagittal), and the coronal plane. These planes divide the skull into four volume quadrants (Q1–Q4) (Figs. 3, 4 and 5). In vertex view, Q1 encompasses the left anterior volume quadrant, which is followed by Q2–Q4 in clockwise orientation. Except for the *30° diagonal difference*, all stereophotogrammetric parameters are measured in the base plane. To analyse the *30° diagonal difference*, an additional measurement plane is needed that is parallel to the base plane and is shifted to the largest horizontal circumference of the head. Automated analyses of these virtual skulls and their reference points were performed with Cranioform Analytics^®^ 4.0 software (Cranioform, Alpnach, Switzerland).

### Statistical analysis

For both the patient and the control group, seven different metric variables were defined and descriptively analysed (see Table 2). Five of these seven variables were well-defined measurement parameters:

- ACAI (Anterior Cranial Asymmetry Index)
- PCAI (Posterior Cranial Asymmetry Index)
- 30° diagonal difference
- Ear shift
- Asymmetry (direct cephalometry, manually measured as CVA)

**Table 2:** Parameters.

	Dimension		Parameter	Unit	Definition
	3D		ACAI	%	Anterior cranial asymmetry index: $$\frac{(larger anterior volume-smaller anterior volume)}{smaller anterior volume}\times 100$$
	3D		PCAI	%	Posterior cranial asymmetry index: $$\frac{\left(larger posterior volume-smaller posterior volume\right)}{smaller posterior volume}\times 100$$
	2D		30° diagonal difference	cm	Length difference between the two diagonals 30° to the y-axis: $longer diagonal-shorter diagonal$
	2D		Ear shift	cm	Sagittal transposition of the tragus points in the base planar compared with in the coronal plane
	2D		CVA	cm	Length difference between the two diagonals of fronto-zygoma – eurion (CVA): $longer diagonal-shorter diagonal$
	3D		|ln(Q4/Q3)|		Absolute value of the logarithmic ratio of the two posterior volumes: $|\mathrm{ln}(\frac{Q4}{Q3})|$
	3D		|ln(Q4Q2/Q3Q1)|		Absolute value of the logarithmic ratio of the multiplied diagonal volumes: $|\mathrm{ln}\left(\frac{Q4Q2}{Q3Q1}\right)|$

In order to quantify asymmetries, pre-analysis consisted of a graphical analysis of scatterplots of a large range of obviously suggestive volumetric relations. Due to the enormous amount of data we will not elaborate these analysis. Finally, two new parameters were identified as potentially appropriate volume parameters:

- |ln(Q4/Q3)|
- |ln(Q4Q2/Q3Q1)|

The five already-established parameters as well as the two new volume parameters were further examined for significant differences between the plagiocephalic group and control group. To compare both groups, a t-test was used in case of standard distribution, and a Mann–Whitney U test was used in case of non-normal distribution.

When examining more than two groups (*control* vs *moderate PP* vs *severe PP*), ANOVA was used in case of standard distribution and variance homogeneity. This variance homogeneity was examined using Levene’s test. After ANOVA, post-hoc tests were performed to examine pairwise differences. Here, we applied the by Games–Howell test for alpha-error accumulation. For variables that showed no standard distribution, a Kruskal–Wallis test was performed to compare the three groups. Again, as post-hoc tests, Mann–Whitney U tests were calculated pairwise, and Bonferroni correction was used for alpha-error accumulation. A significance level of p < 0.0125 (0.05/4) resulted.

To further determine the best parameter for qualitatively distinguishing between the three groups (*control* vs *moderate PP* vs *severe PP*), a receiver-operating-characteristics (ROC) analysis was calculated. As this ROC analysis is a dichotomous procedure, two separate ROC analyses had to be calculated.

The first analysis examined parameter quality as a classifier for differentiating between the control group and the patient group (*moderate* and *severe PP*).

The second analysis examined the differences between the control group (*no PP*), the *moderate PP* group, and the *severe PP* group in comparison with two-dimensional parameters. For each parameter, a curve shows sensitivity and specificity in possible cut-off values. The area under the curve (AUC) determines the area between the curve and the reference line that corresponds to the angle bisector of the coordinate system. An AUC of 1 is the maximum achievable value and identifies a perfect classifier. An AUC of 0.5 (a curve close to the angle bisector) is categorised as an accidental result.

As |*ln(Q4Q2)/(Q3Q1)|* proved to be the best volume parameter (highest ROC) after ROC analysis, we set new cut-off values for this parameter that defined the three groups of *no PP*, *moderate PP*, and *severe PP*. Cut-off values were the values with the highest possible sensitivity along with the highest possible specificity (Youden’s index). These calculated thresholds enabled a new group variable to be set, which was compared with the previous gold standard of *30° diagonal difference*. Thus, a new categorisation of the patients in the three groups resulted from using the volume parameter of |*ln(Q4Q2/Q3Q1)|*.

### Informed consent

The informed consent analysing the 3D-Photoscans obtained from study participants was written by the participants’ parents.

## Results

### Differentiating between no PP and moderate + severe PP

The t-test and Mann–Whitney U test that were performed to evaluate the difference between the patient group and the control group resulted in significant differences for all examined parameters (p < 0.05).

For every parameter in which significant differences between these groups resulted, pairwise comparisons were performed. The parameters and their corresponding post-hoc analysis—which were examined using ANOVA (p < 0.0125) and the Kruskal–Wallis test (p < 0.05)—also revealed significant differences in all comparisons.

### ROC AUC analysis of no PP compared with that of moderate + severe PP

**Figure 6:**
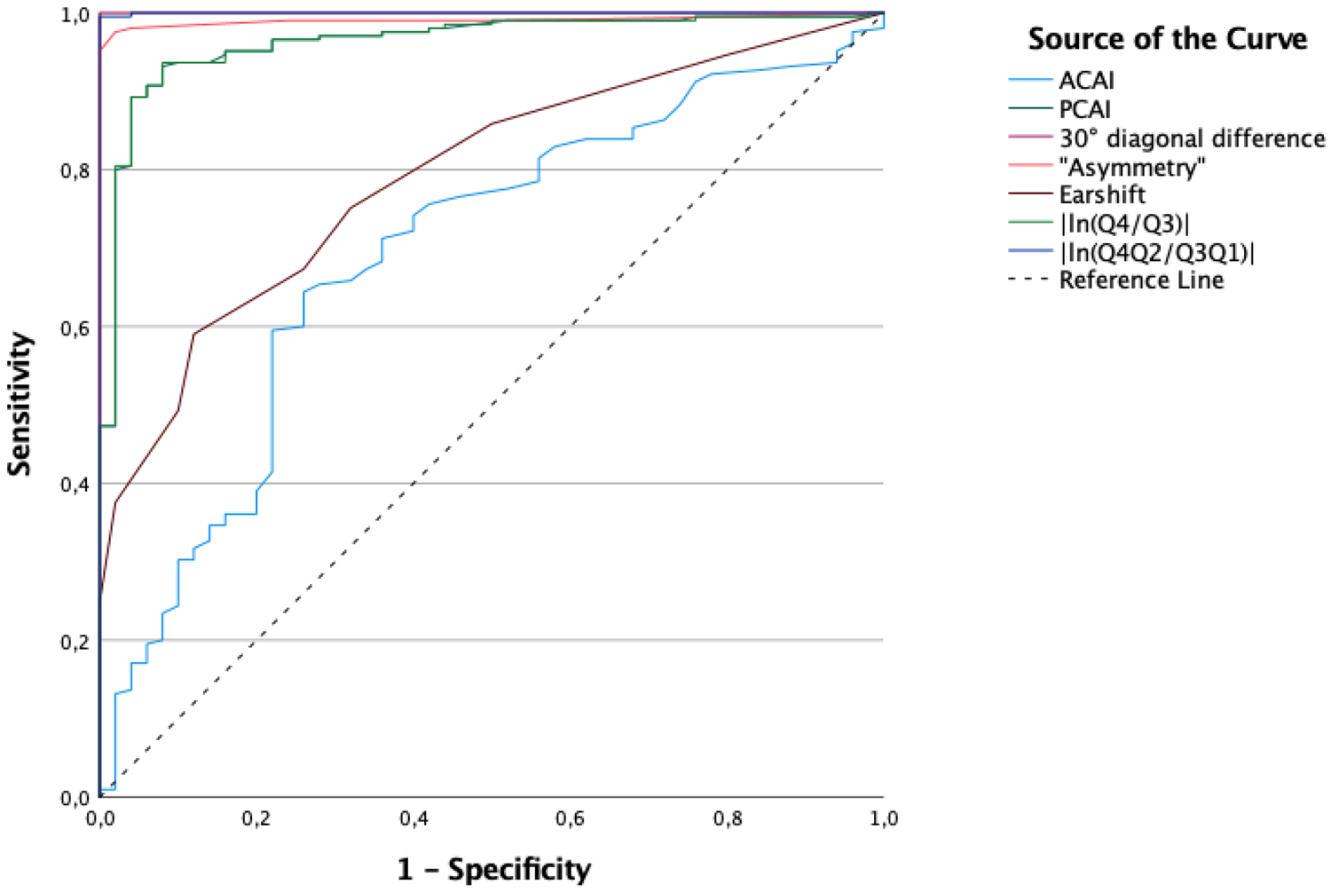
ROC curves of ‘no PP’ compared with those of ‘moderate + severe PP’.

The comparison of the control group (*no PP*) with *moderate* and *severe PP* is shown in a ROC curve for each parameter. These curves visualise sensitivity and specificity for different cut-off points (Fig. 6). The steepest curve was found for *30° diagonal difference* and for the volume parameter of *|ln(Q4Q2/Q3Q1)|.* The flattest curve was found for the parameter of *ACAI*, and a medium-sloped curve was found for the parameter of *ear shift*. The calculated AUCs are outlined in Table 3.

**Table 3:** ROC AUC value of ‘no PP’ compared with that of ‘moderate + severe PP’.

Parameter	AUC
ACAI	0.694
PCAI	0.963
30° diagonal difference	1.000
Ear shift	0.793
Asymmetry	0.991
|ln(Q4/Q3)|	0.963
|ln(Q4Q2/Q3Q1)|	1.000

Here, the parameter of *30° diagonal difference* and the volume parameter of *|ln(Q4Q2/Q3Q1)|* yielded a maximum AUC of 1, and the parameter of *ACAI* had the lowest value (AUC = 0.694). With an AUC of 0.991, the parameter of *asymmetry* (measured manually) almost reached the maximum AUC of 1.

### ROC AUC analysis of severe PP compared with that of no + moderate PP

**Figure 7:**
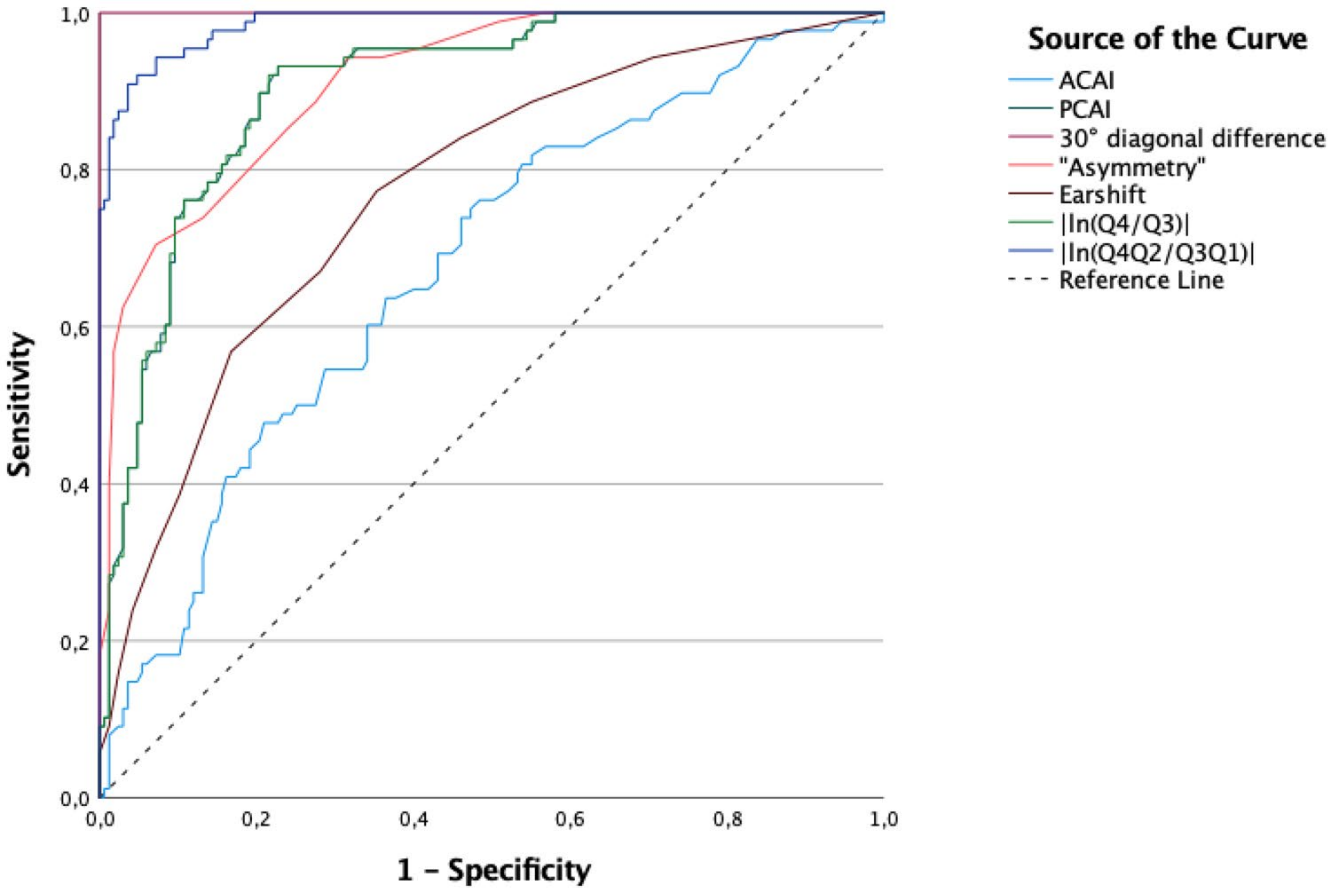
ROC curves for ‘severe PP’ compared with those of ‘no + moderate PP’.

The steepest curve can be seen within the parameter of *30° diagonal difference* (Fig. 7). The volume parameter of *|ln(Q4Q2/Q3Q1)|* also yielded a steep curve. A medium-sloped curve was found for the parameter of *ear shift*. The parameter of *ACAI* yielded a flat curve.

The calculated AUCs are outlined in Table 4. A maximum value of 1 was achieved by the parameter of *30° diagonal difference*, followed by *|ln(Q4Q2/Q3Q1)|*, with an AUC of 0.986. An AUC of 0.905 was found for the parameter of *PCAI*. The volume parameter of *|Q3–Q4|* achieved an AUC of 0.914, and the parameter of *asymmetry* achieved an AUC of 0.913.

**Table 4:** ‘Severe PP’ compared with ‘no + moderate PP’.

Parameter	AUC
ACAI	0.676
PCAI	0.905
30° diagonal difference	1.000
Ear shift	0.771
Asymmetry	0.913
|ln(Q4/Q3)|	0.905
|ln(Q4Q2/Q3Q1)|	0.986

### Threshold for the volume parameter of *|ln(Q4Q2/Q3Q1)|*

In order to set the thresholds (cut-off points), we determined the coordinate pairs of the volume parameter of |*ln(Q4Q2/Q3Q1)|* in Figs. 6 and 7 that showed the highest Youden’s index *J (*$J=sensitivity+specificity-1$). Graphically, *J* is the maximum vertical distance between the ROC curve and the diagonal reference line^23^.

Corresponding cut-off values for the volume parameter of |*ln(Q4Q2/Q3Q1)|* are shown in Table 5, and the new classification for graduating PP results is presented in Table 6.

**Table 5:** Threshold definition for |ln(Q4Q2/Q3Q1)|.

PP	Cut-off value	Sensitivity	1 − specificity	Specificity	AUC
Moderate	0.0743	0.9952	0.0000	1.0000	1.0000
Severe	0.2412	0.9451	0.0769	0.9231	0.9860

**Table 6:** PP classification using the volume parameter of |ln(Q4Q2/Q3Q1)|.

PP	Cut-off value
No	< 0.0743
Moderate	0.0743–0.2412
Severe	> 0.2412

### New classification of subject groups using the volume parameter of |*ln(Q4Q2/Q3Q1)*|

**Figure 8:**
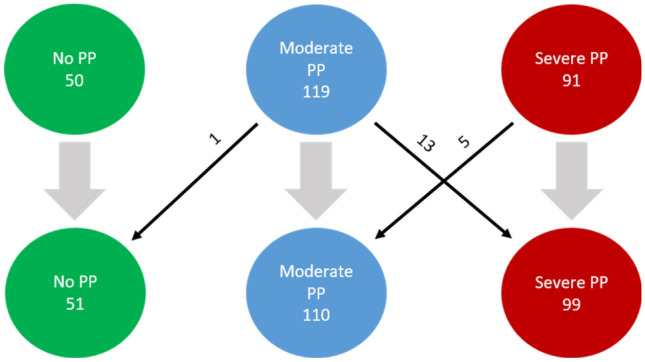
Rearrangement within ‘non-PP’ and ‘PP’ groups.

When applying the new cut-off values defined in Table 5, a quantitative rearrangement within the groups resulted (Fig. 8). The group of *no PP* (control group) increased by one infant from the *moderate PP* group. When using the volume-based classification, 13 infants from the *moderate PP* group were shifted to the *severe PP* group. However, five infants from *severe PP* group were shifted to the *moderate PP* group.

In sum, the *severe PP* group increased by eight infants, the *moderate PP* group decreased by nine infants, and one infant was moved to the *control* group (Fig. 8).

## Discussion

Different measurement- and classification systems are used for quantifying PP, which is the most common skull deformity in infants. The majority of the gathered data on PP and the resulting classifications use two-dimensional measurement methods, as is the case with the classification by Moss and with that by Loveday et al.^6,10^. The use of this variety of methods is problematic from a clinical and scientific point of view because it complicates both the scientific discourse and the ability to reach an objective therapy decision.

Direct cephalometry—which is performed manually on the baby’s skull with a measuring tape and calliper—is common, although its reliability remains the subject of ongoing discussion^1,8,24,25^. Compared with this direct measurement of pure distances, stereophotogrammetry enables 3D data to be acquired and demonstrates a higher reliability as well as a very low artefact rate^12,17,18,26–28^. As a radiation-free procedure, stereophotogrammetry allows for repeated scans in the course of therapy and follow-up.

It is a reliable examination method and has good intra- as well as inter-examiner reliability, especially regarding the 3D parameters^29^.

However, it is still common practice to extract only 2D parameters from this 3D stereophotogrammetric set of data. This reduction of a three-dimensional object by using only two-dimensional parameters has a significant potential for error. In 2010, Lipira et al. demonstrated the extent to which even small deviations in the measurement plane can negatively affect the circumference and the measured distances^14^.

As shown in the present text, three-dimensional measurement methods have the potential advantage of more precisely representing the skull in its entirety^19,20,30,31^.

However, no appropriate 3D volume parameters or resulting indices have yet been described. Existing 3D parameters—such as *ACAI* or *PCAI*, which are used to categorise severities of PP—have turned out to be of limited suitability for clinical practice as they only represent the frontal or occipital volume quadrant and do not consider the entire three-dimensionality of the skull.

To the authors’ knowledge, none of the common, above-mentioned 2D or 3D classification have been further validated for clinical use thus far. One scope of our study was therefore to establish a suitable three-dimensional asymmetry index for clinical and scientific dialogue.

In addition, we generated a new classification using 3D stereophotogrammetric volume data for the first time by taking the three-dimensionality of the skull into full account. For this purpose, we performed a systematic statistical workup that investigated children both with and without PP.

Initial tests yielded highly significant distinctions between the patient group and the control group, thereby allowing for a clear differentiation based on the specific chosen parameters. To ensure comparability to the existing literature, we performed our analyses and created a classification system based on *no*, *moderate*, and *severe PP*, which is in line with Moss and Mortenson et al.^6,11^. Due to the widespread usage of the *30° diagonal difference* described by Loveday et al.^10^, our measurements also originated from this gold standard. Separating the PP group by the *30° diagonal difference* caused the ROC analysis that had been performed for this 2D diagonal parameter resulted in the highest possible value of 1, as shown in Figs. 6 and 7.

Prior to generating the presented data, we had performed a comprehensive pre-analysis in order to identify potential appropriate volume parameters. The two most promising volume parameters—*|ln(Q4Q2/Q3Q1)|* and |ln|*Q3–Q4*|—were depicted and evaluated in our study in order to facilitate a 3D-based classification of PP for the first time. In our ROC analysis parameter, *|ln(Q4Q2/Q3Q1)|* performed superior to the parameter of |*Q3–Q4*|, and we therefore agreed to use |*ln(Q4Q2/Q3Q1)|* for further statistical investigations. As |*ln(Q4Q2/Q3Q1)|* is a bulky label, we instead use the term *3D asymmetry index*.

To differentiate between infants *without* and *with moderate to severe PP*, the volume parameter *3D asymmetry index* with an AUC of 1 represented the maximum possible value. When differentiating between infants with *moderate PP* and infants with *severe PP*, the AUC was almost 1 (0.986). However, it still yielded the highest value, which means that no other 2D or 3D parameter had higher values in its respective group. The *30° diagonal difference* of course scored an AUC of 1 (as shown above) because it defined the groups.

It should also be noted that the manual measurements with a calliper that were used to differentiate between *moderate* and *severe PP* also yielded very good results, with an AUC of 0.914. In differentiating between *no PP* and *moderate* + *severe PP*, these measurements even reached an AUC of 0.991. This finding highlights the strong results of this measurement method when used by experienced examiners, as has been reported in other studies^11,25^.

In their recently published report, Kato et al. evaluated the degree of severity in children with PP using 2D and 3D parameters. They could show that the severity assessment differs between 2 and 3D evaluation in one of six children. For 3D evaluation, they observed the anterior and posterior volume quadrants separately^29^. As shown above the comparison of both frontal volume quadrants (*ACAI*) resulted in considerably worse results than were found for all other parameters and proved to not be suitable. Clinical examination revealed that the extent of the frontal bossing did not correlate with the extent of the occipital flattening.

Although mainly the occipital region is affected in PP, the single juxtaposition of both occipital volume quadrants (*PCAI*) also yielded insufficient values to describe an existing asymmetry. This finding reveals that a single analysis of volume changes—either in the frontal or occipital quadrants—obviously does not solve the problem of reliably classifying PP.

Only a few studies have investigated *ear shift*. Kluba et al. demonstrated that only a weak correlation exists between the extent of the asymmetry and an ear shift^32^. In our own published data, we have also described *ear shift* as a weak parameter for classifying PP^33^. The conclusion of the presented analysis again stresses the notion that *ear shift* is not suitable as single differentiation criterion in PP. These results can be confirmed by clinical observations made by experienced examiners^32^.

Using the new volume parameter *3D asymmetry index*, we established a new classification of PP using threshold determination. The *30° diagonal difference* for differentiating between *mild to moderate PP* and *moderate to severe PP—*which is the actual gold standard in the existing literature—was maintained purposely for better comparability and easier applicability. After reclassifying the patients into three groups using the newly determined thresholds, we observed only minor shifts in the strength of each group when using the *3D asymmetry index* as compared with the 2D parameter of *30° diagonal difference*.

Our parameter *3D asymmetry index* advances the development of the common classification systems used for PP. By describing *no* (< 0.0743), *moderate* (0.0743–0.2412), and *severe* (> 0.2412) degrees of PP, this index can be used as part of a new classification system that considers the entire three-dimensionality of the skull.

In accordance with existing studies, we included infants with comparable age and sex distribution. Although the number of included infants allowed for good statistical conformity, further validation with larger numbers in multicentre studies is needed.

As *30° diagonal difference* is most commonly used to classify PP in present literature, we chose this parameter in the present study as well. Consequently, *30° diagonal difference* reached the maximum possible AUC-value of 1. This means, that *3D asymmetry index* can only be as good as *30° diagonal difference* due to this methodology, which is a limitation in this study.

The comparison of relative values (ratios) in 3D parameters to absolute values in 2D parameters is a methodological limitation in this study. As mentioned above, we chose the *30° diagonal difference* since it is the most commonly used parameter in literature as well as in daily clinical practice.

As a real three-dimensional parameter, a *3D asymmetry index* might serve as a valuable basis for further studies that take clinical appearance and objective measurements into account, which has not yet been performed in the existing literature.

## Conclusion

A variety of different measurement methods and values exist for classifying PP, the majority of which use two-dimensional measurements. Our analysis reveals that direct cephalometry—performed by an experienced examiner—already has a high reliability in two-dimensional classification of PP. Furthermore, we were able to identify problems with the described 3D parameters of *PCAI* and *ACAI* in clinical use.

Using a combination of 3D analysis quantification, we developed the new volume parameter of a *3D asymmetry index*, which proved to be highly suitable for describing and classifying PP three-dimensionally for the first time.

## Data Availability

The datasets used and/or analysed during the current study available from the corresponding author on reasonable request.
